# Terahertz Fingerprint of Monolayer Wigner Crystals

**DOI:** 10.1021/acs.nanolett.1c04620

**Published:** 2022-01-20

**Authors:** Samuel Brem, Ermin Malic

**Affiliations:** † Department of Physics, Philipps University, 35037 Marburg, Germany; ‡ Department of Physics, Chalmers University of Technology, 41258 Göteborg, Sweden

**Keywords:** Wigner crystal, 2D materials, Terahertz spectroscopy, transition metal dichalcogenides, density matrix theory

## Abstract

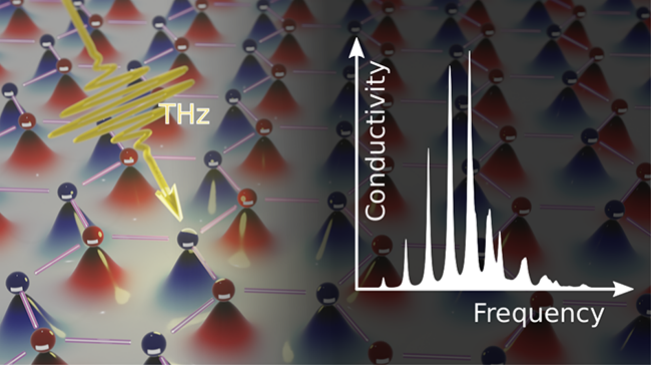

The strong Coulomb interaction in monolayer semiconductors represents a unique opportunity for the realization of Wigner crystals without external magnetic fields. In this work, we predict that the formation of monolayer Wigner crystals can be detected by their terahertz response spectrum, which exhibits a characteristic sequence of internal optical transitions. We apply the density matrix formalism to derive the internal quantum structure and the optical conductivity of the Wigner crystal and to microscopically analyze the multipeak shape of the obtained terahertz spectrum. Moreover, we predict a characteristic shift of the peak position as a function of charge density for different atomically thin materials and show how our results can be generalized to an arbitrary two-dimensional system.

At low temperatures, electrons arrange in a crystal lattice^1^ in order to minimize their repulsive energy, which represents one of the most intriguing quantum phase transitions. Ever since their prediction, the experimental realization of these Wigner crystals (WCs) has remained challenging, considering that the prerequisite of a dominating Coulomb versus kinetic energy requires very low temperatures and charge densities. Therefore, the first realizations of WCs^2−4^ have used strong magnetic fields and quasi two-dimensional (2D) quantum well systems in order to quench the kinetic energy via Landau quantization. The advances in the research on transition-metal dichalcogenide (TMD) monolayers^5−7^ have delivered new opportunities to realize electron crystallization. Several recent studies^8−12^ have reported insulating states at fractional fillings of flat moiré bands,^13−15^ which arise in the superlattices of twisted homobilayer and heterobilayer systems. However, similar to the application of magnetic fields, these so-called generalized Wigner crystals heavily rely on the external moiré potential stabilizing the lattice structure and can therefore not be considered as intrinsic electronic phases.

In contrast, TMDs in their monolayer form already represent a unique platform to achieve electron crystallization, as they exhibit large effective masses and a strong Coulomb interaction leading to prominent exciton physics.^16,17^ Smoleński et al. have recently demonstrated signatures of a WC formed in hBN-encapsulated MoSe_2_ monolayers^18^ at electron densities of up to *n* = 3 × 10^11^ cm^–2^ without any external fields. As an indicator for the crystallization, the authors have used the emergence of an additional peak in the reflectance spectrum at visible frequencies. This peak stems from excitons with finite center-of-mass momentum that couple to the light cone via Bragg scattering at the WC. Other rather indirect confirmations of the WC, such as a decrease in the conductivity, also have been applied to verify other WC systems.^2−4,8,10−12^ However, a direct, more reliable and conclusive experimental proof of the WC, such as a direct spectral signature of the internal structure of the crystal, could not be provided so far.

In this work, we propose to exploit terahertz radiation to directly probe the internal quantum transitions of the WC in TMD monolayers, as illustrated in Figure 1. We have developed a fully quantum mechanical model of the interaction of monolayer WC with low frequency light. Our model is based on the many-particle density matrix formalism, where the Hamiltonian of interacting electrons is transformed to the self-consistent eigenbasis of the WC on a Hartree–Fock level. Consequently, we analyze the linear optical response of the system resulting from transitions between different bands of the electron crystal.

**Figure 1:**
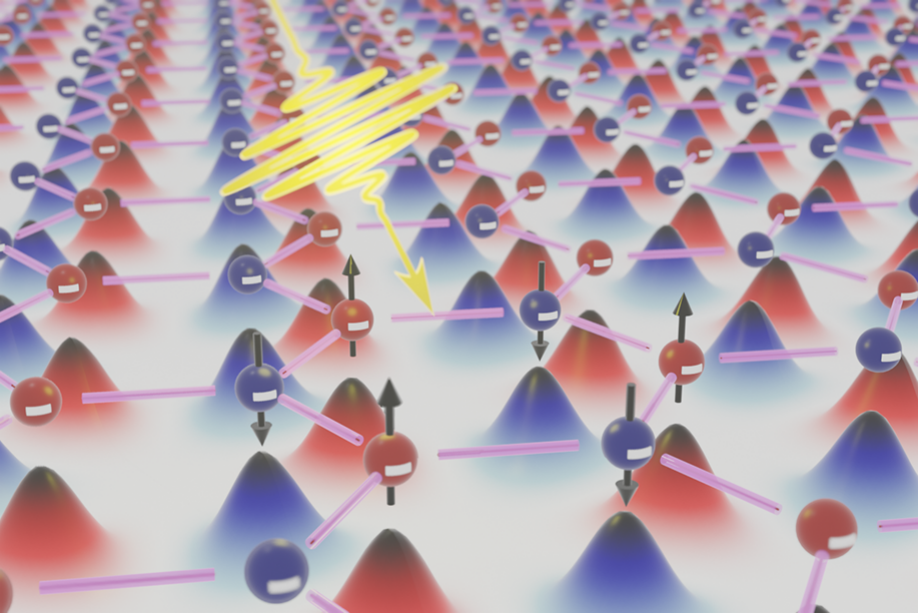
Sketch of the 2D Wigner crystal with a honeycomb lattice and alternating spin polarization. The colored curves underneath the particles illustrate their wave functions. A low-energy light field can probe the internal transition of the Wigner crystal toward excited quantum states to provide a conclusive proof of the phase transition.

We predict the emergence of a series of terahertz resonances whose position is a function of electron density and its characteristic peak sequence can be explained by the nature of the involved excited quantum states. Consequently, the predicted low frequency response can be used as a direct probe of the Wigner crystallization in arbitrary 2D systems of interacting fermions. We apply our microscopic model to determine the material realistic response of hBN-encapsulated WSe_2_ monolayers without any open parameters and explain the characteristic response shape with the help of our microscopic model. We predict that the central energy of the internal quantum resonances shifts from ∼4 meV at 10^9^ cm^–2^ to 24 meV at 8 × 10^10^ cm^–2^. Moreover, we demonstrate that an enhanced effective mass and reduced dielectric screening, such as in MoSe_2_ on SiO_2_, can push the WC resonances up to 100 meV. Finally, we also present a unitless study for ideal 2D systems, which can be rescaled to arbitrary fermionic systems.

## Microscopic Mean Field Theory

Previous numerical studies on WCs based on quantum Monte Carlo methods^19^ have demonstrated that the low-density crystal phase is characterized by small correlation energies and that the WCs properties are well-reproduced within a Hartree–Fock description of the Coulomb interaction.^20^ To obtain access to the optical response of the WC, we start with the Hamilton operator of interacting electrons,
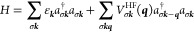
1which can, e.g., be related to the free charge carriers within *n*(*p*)-doped TMD monolayers, such that *a*_σ***k***_^(†)^ creates/annihilates electrons (holes) in the lowest conduction band (highest valence band) with spin up at the *K* point (σ = *↑*) or spin down at the −*K* point (σ = *↓*) and momentum **k**. For low temperature and densities, the electron dispersion ε_**k**_ is determined by an effective mass m_*_.^21^ The Coulomb interaction is treated in a mean field approximation^20,22^ via the nonlocal Hartree–Fock potential,
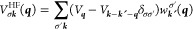
2depending on the Fourier transform of the Coulomb potential *V*_**q**_, which includes the dielectric environment of the TMD monolayer through the Rytova–Keldysh potential.^23,24^ Moreover, the mean field potential also is dependent on the charge distribution entering via the Fourier transform of the Wigner distribution *w*_***k***_^σ^(***q***) = ⟨*a*_σ***k***+***q***_^†^*a*_σ***k***_⟩. The Hamiltonian in eq 1 is now diagonalized via the basis transformation *A*_λσ***k***_^†^ = ∑_***G***_*u*_σ***k***_^λ*^(***G***)*a*_σ,***k***+***G***_^†^, where we sum over the reciprocal lattice vectors **G** of the WC. Here, *u*_σ***k***_^λ^(***G***) is the complete set of Bloch functions fulfilling the periodic Hartree–Fock equation (see the Supporting Information (SI)). The latter is solved iteratively until a self-consistent solution is found, starting with a randomized charge distribution. Hence, in the new basis describing WC electrons the Hamiltonian reads
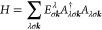
3where the self-consistent solution requires that the lowest band of the WC (λ = 0) is completely filled (one electron per WC unit cell) and the rest is empty, i.e., *f*_λσ***k***_ = ⟨*A*_λσ***k***_^†^*A*_λσ***k***_⟩ = δ_λ0_.

Having determined the energy spectrum of WC electrons *E*_σ***k***_^λ^ and their wave functions *u*_σ***k***_^λ^(*G*), we can transform the electron-light interaction into the Wigner basis and compute the dynamics of the WC using the Heisenberg’s equation of motion.^25,26^ In particular, assuming a weak perturbation through the electromagnetic field, we can derive the linear response of the system, which, e.g., can be specified via the diagonal components of the linear optical conductivity tensor reading:
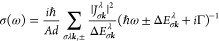
4where we have introduced the monolayer thickness *d*,^27^ the normalization area *A*, and a phenomenological dephasing constant Γ. The oscillator strength of transitions between different WC sub-bands with energy Δ*E*_σ***k***_^λ^ = *E*_σ***k***_^λ^ – *E*_σ***k***_^0^ for *ê*-polarized light is given by the current matrix element *J*_σ***k***_^λ^=*e*_0_*ℏ*/m_*_∑_**G**_*u*_σ***k***_^λ*^(***G***)***G***·*êu*_σ***k***_^0^(***G***). All details of the derivation and the used parameters are given in the SI. The microscopic model described above is not specific to TMD monolayers and can be applied to arbitrary 2D electron system. In principle, a similar approach can be used to compute the optical response also in generalized WCs within twisted bilayers.

## Internal Quantum Structure of WCs

**Figure 2:**
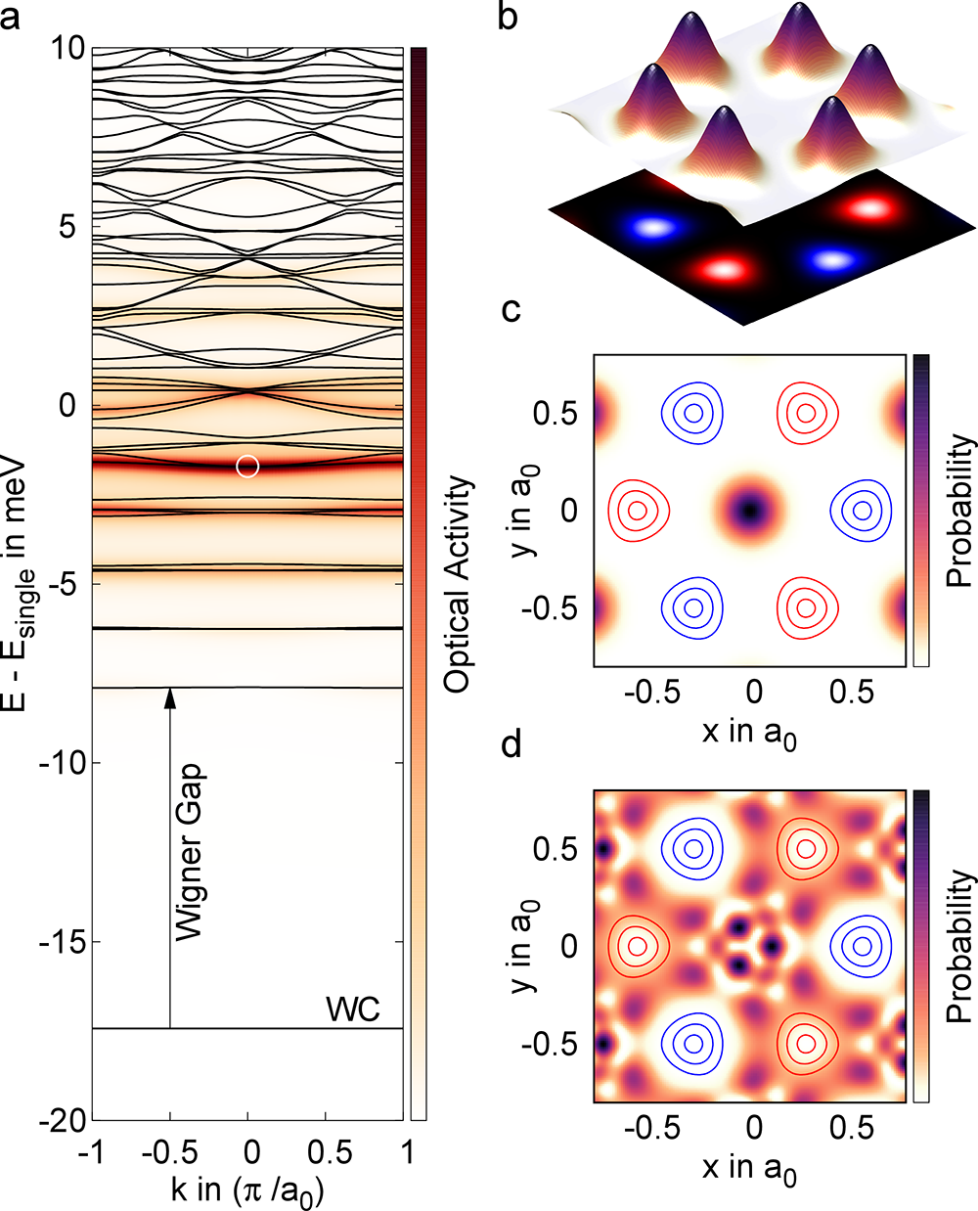
Internal quantum structure of the WC in *p*-doped, hBN-encapsulated WSe_2_ at *r*_s_ = 50. (a) Band structure of the WC, where only the lowest band is fully occupied. The background color illustrates the oscillator strength for optical transitions from the ground state. (b) The surface plot shows the charge density, whereas the projection contains the spin polarization (*↑* is red, *↓* is blue). Also shown are the wave function at **k** = 0 of the (c) first excited state and (d) the state with the largest oscillator.

Now, we apply the developed model to the exemplary system of *p*-doped, hBN-encapsulated WSe_2_ monolayers at a density of *n* ≈ 3 × 10^10^ cm^–2^ (*r*_s_ = 50), i.e., deep within the crystalline phase that was predicted to melt at *r*_s_ = 30–40.^28−30^ The temperature is assumed low enough such that only the lowest band of the WC is occupied and the thermal occupation of the excited states can be neglected. Moreover, we focus on the spin-unpolarized (antiferromagnetic) phase, since we assume that the charge density is controlled via an electrostatic gate. As a result, the Wigner crystal is in a chemical equilibrium with an unpolarized charge reservoir. Apart from the WC geometry, the main conclusions qualitatively also hold for spin (valley)-polarized systems.^31^Figure 2 shows the computed band structure for electrons with σ = *↑* (on a cut through the WCs Brillouin zone) as well as the wave functions of the monolayer WC. The band structure is illustrated in Figure 2a, where the background has been color-coded based on the optical activity,
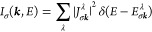
We find a completely flat ground state at ∼17 meV below the edge of the single particle dispersion. We also observe a series of flat excited states, where the first is separated from the ground state by a Wigner gap of ∼10 meV. With the increasing band index, the excited states become more dense and dispersed. In order to interpret this result, we investigate the underlying nature of these quantum states and their respective wave functions.

The fully occupied ground state is, in fact, the WC itself, i.e., the corresponding wave functions are eigenstates of the Coulomb potential created by their own collective charge distribution. Here, the charge density (Figure 2b) exhibits strongly localized peaks ordered in a honeycomb lattice^32^ with alternating spin polarization (red and blue projections). Moreover, the localized charge distributions exhibit a significant triangular warping, which is usually neglected in variational approaches.

In contrast to the self-consistent ground state, the excited states are unoccupied and therefore do not contribute to the nonlocal Hartree–Fock potential (see the SI). Figure 2c shows the wave function of the first excited state at **k** = 0, whereas the contours indicate the peaks of the *↑* (red) and *↓* charge density (blue) from Figure 2b. In order to minimize the repulsive Coulomb energy, the excited state is strongly localized in the pockets between the charge peaks. Consequently, the wave function overlap with the ground state is small, which is reflected by the weak optical activity of the first excited state. With the increasing band index, we first find several other localized states with increasing kinetic energy and growing spatial extent.^15^ Consequently, the localized orbitals of higher-order bands begin to overlap at higher quantum indices and we find dispersed bands at high energies corresponding to scattering states. Figure 2d shows the excited state with the largest optical activity for transitions from the occupied ground state. As result of its delocalized character and the beneficial exchange interaction between equally spin-polarized electrons, the overlap with the ground state (red contours) is large, resulting in a strong current matrix element. The proposed detection mechanism via low-energy excitations has previously been applied to WCs formed in GaAs quantum wells in strong magnetic fields, where a single peak has been observed at microwave energies,^33,34^ the so-called pinning frequency.^35^ In contrast, our microscopic model predicts that zero-field monolayer Wigner crystals should exhibit an entire series of resonances at much larger frequencies.

With the knowledge of the internal WC quantum structure, we can now consider the dynamics of the system under external perturbations, e.g., the absorption of light.

## Optical Fingerprint of WCs

**Figure 3:**
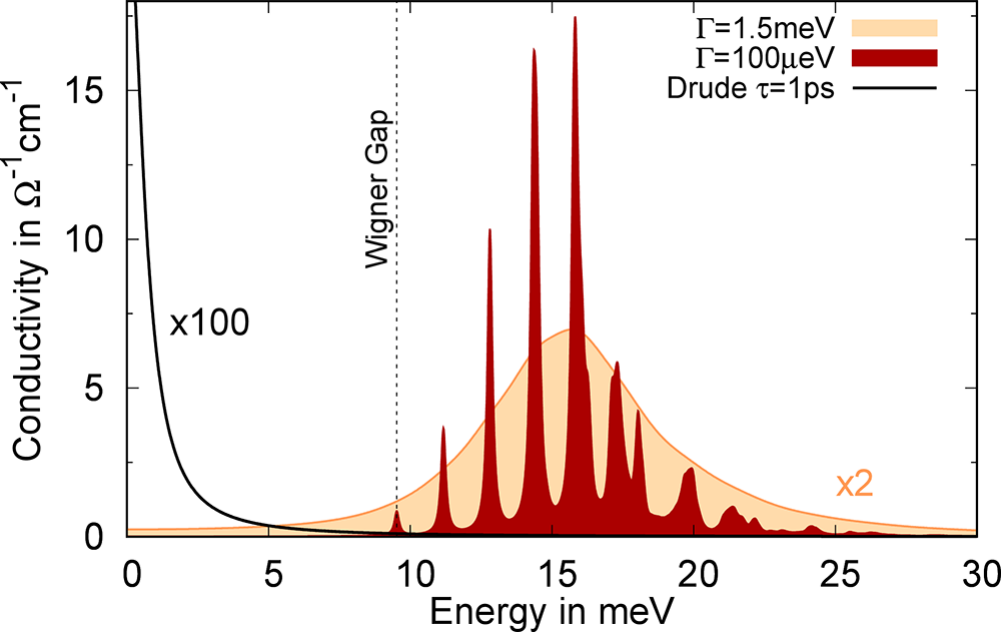
Terahertz fingerprint of the Wigner crystal. The real part of the optical conductivity exhibits a characteristic series of internal quantum transitions, whose amplitude sequence can be explained by the nature of the involved excited states. Note that the strongly broadened response signal has been scaled by a factor of 2, whereas the Drude signal of free electrons (black line) has been multiplied by 100.

Now, we evaluate the linear optical conductivity from eq 4 in the energy range of the WCs interband transition. Figure 3 illustrates the real part of the resulting spectrum for two exemplary line widths. For the narrower line width of Γ = 0.1 meV, the spectrum exhibits a distinguished multipeak structure with a characteristic sequence of peak amplitudes. The series of resonances starts at the energy of the Wigner gap (cf. Figure 2a), reflecting the transition to the first excited state, which has a weak oscillator strength, as discussed above. The subsequent series of peaks corresponds to the transition to higher-order excited states, whose oscillator strength increases until a maximum is reached. This is a result of the growing kinetic energy of the excited states with increasing band index. With the increasing orbital size of the excited states, which are mostly localized in the pockets between the WC electrons, their overlap with the ground-state electrons becomes enhanced. After a maximum is reached for the excited state illustrated in Figure 2d, the oscillator strength decreases again. Here, the final states momentum spectrum *u*_σ**k**_^λ^(***G***) begins to move toward large **G** vectors, corresponding to scattering states with a large kinetic energy. Consequently, the momentum space overlap determining *J*_σ***k***_^λ^ shrinks.

The calculated conductivity, on the order of 10 Ω^–1^ cm^–1^, corresponds to a rather low overall absorbance of the monolayer in the range of α = *dRe*(σ)/(*c*ϵ_0_) ≈ 10^–4^, which is due to the nanometer thickness of the sample. However, comparably weak low-frequency responses stemming from intraexcitonic transitions have already been used to measure time-resolved exciton dynamics in TMD monolayers.^36^

## Density Dependence

**Figure 4:**
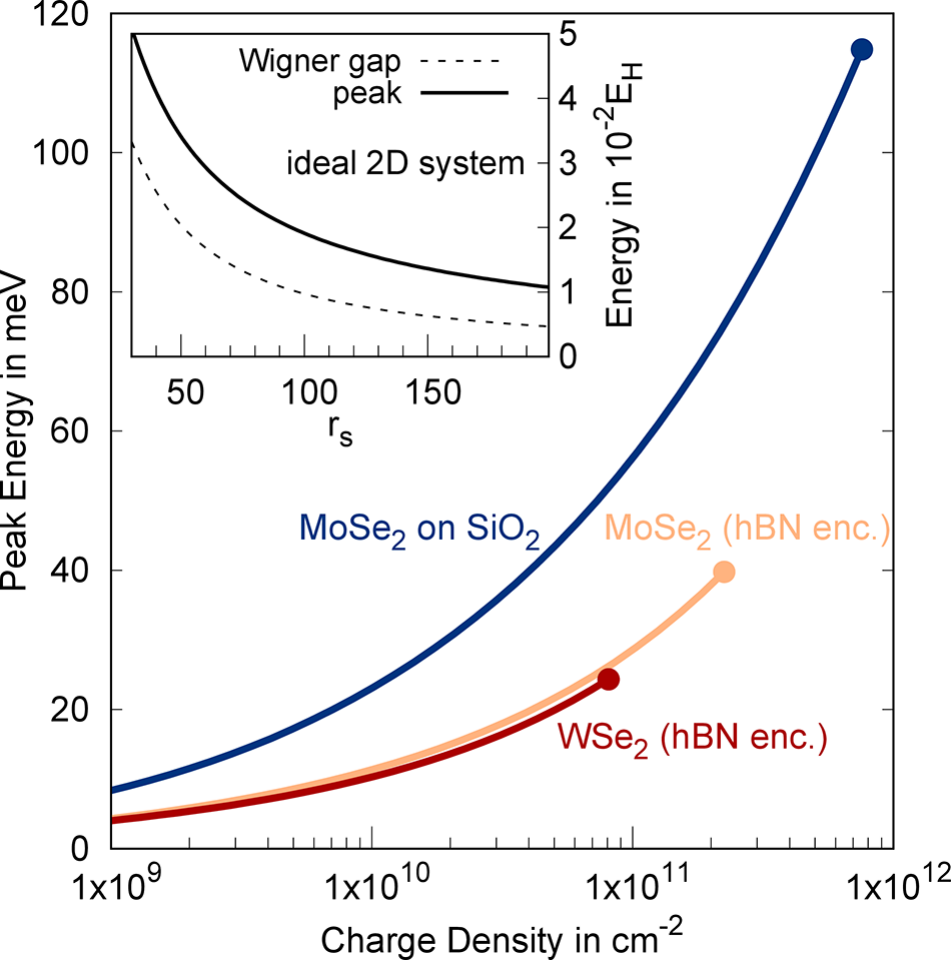
Central energy of the optical response signal as a function of the hole density for different monolayers/substrates. The inset shows the result for an ideal 2D system, where the only system parameter is given by *r*_s_ and the energy scale is given in units of effective Hartree.

Now, we vary the charge density and investigate how the spectral response shifts as the lattice parameter of the WC, , is changed. To this end, we consider the center of the spectral response defined as ω_peak_ = ∫ωσ(ω)dω/∫σ(ω)dω, where the integrals are only performed over positive frequencies. Figure 4 shows the center energy of the optical response as a function of density for different TMD monolayers. The filled dots represent the values at *r*_*s*_ = 30, such that for higher densities quantum melting of the WC is expected. For all three illustrated systems we consider p-doping, since the valence band in TMDs has a significantly larger mass then the conduction band, which enables larger critical densities before quantum melting sets in. Figure 4 illustrates that the optical response shifts significantly to higher energy at enhanced charge densities, which can serve as a clear indicator that the observed peaks indeed stem from transitions of the WC. With increasing density, the charge density peaks are more closely spaced, which enhances the repulsive Coulomb energy for the excited states and therefore increases the transition energies. As a result, we also find that the transition energies for samples on SiO_2_ are significantly larger, since the Coulomb interaction is much less screened by the substrate. In addition to the spectral shift of the center energy, the amplitude of the optical response also increases with growing densities, since the density of optically active particles is enhanced.

We have additionally performed calculations for an ideal 2D system with *V*_***q***_ ∝ 1/***q***, which reproduces the results for TMD monolayers at small densities. For an ideal 2D system, the only relevant system parameter is given by  with the effective Bohr radius being defined as *a*_B_ = 4πϵ_0_ϵ*ℏ*^2^/(m_*_e_0_^2^). Using this density scale, the energies can be given in terms of effective Hartree *E*_H_=*ℏ*^2^/(m_*_a_*B*_^2^), such that the results given in the inset of Figure 4 can be rescaled to arbitrary 2D system. The inset furthermore demonstrates that the overall width of the optical response Δ*E* ≈ *E*_peak_ – *E*_gap_ also grows with increasing density, since the distance between excited states becomes larger.

Finally, we want to note that the susceptibility derived in our work results from an oscillating polarization current as a response to the electromagnetic field. Since the oscillating electrons (holes) are forming the WC crystal itself, the optical resonances found in our work could be interpreted in a classical picture as the excitation of long-range phonon modes of the WC.^32^ Moreover, we want to emphasize that the applied model is only valid for weak excitation conditions, i.e., assuming a negligible population of the excited states. A lower bound for the critical power density of the exciting laser can be obtained by estimating a lower bound for the lifetime of excited states. Assuming that the lifetime is shorter than τ = 1 ns, the weak excitation regime can be specified by a power density of *I* ≪ *n*(*E*_1_ – *E*_0_)/τ, which for the systems studied in Figures 2 and 3, yields *I* ≪ 50 mW/cm^2^. For stronger excitation powers, we expect optical nonlinearities to occur that could even result in a destruction of the WC.

In conclusion, our study demonstrates that the terahertz response of monolayer Wigner crystals can be used as an unambiguous fingerprint, exhibiting a characteristic sequence of internal quantum transitions. The inherent spectral shift of resonance frequencies with the charge density further represents a strong benchmark for the crystallization. We have predicted the peak positions for different TMD monolayers, as well as other 2D fermion systems. The presented results will guide future experiments toward the detection of Wigner crystallization and the developed approach can be further exploited to theoretically study the interaction dynamics in pure as well as generalized Wigner crystals in twisted bilayers.

## Supporting Information Available

Transformation of Hartree–Fock Hamiltonian into Wigner basis; material-specific parameters and interaction matrix elements; derivation of the optical response (PDF)
