# Supplementary material for: Terahertz Fingerprint of Monolayer Wigner Crystals
Source: Nano Lett. 2022 Jan 20;22(3):1311–5. doi: 10.1021/acs.nanolett.1c04620 (PMC8832488; doi:10.1021/acs.nanolett.1c04620)
Supplement: Supplementary file 1 — nl1c04620_si_001.pdf [file nl1c04620_si_001.pdf]

Supplementary Information
Terahertz fingerprint of monolayer Wigner crystals

Samuel Brem[1*] and Ermin Malic[1,2†]
[1]*Department of Physics, Philipps University, 35037 Marburg, Germany and*
[2]*Department of Physics, Chalmers University of Technology, 41258 Gothenburg, Sweden*

I. HARTREE-FOCK MODEL OF WIGNER CRYSTALS

In this work, the properties of the Wigner crystal (WC) are described on a mean field level of theory, i.e. the Coulomb interaction is reduced to an effective single-particle potential given by the Hartree-Fock (HF) approximation [1, 2]. The HF-Hamiltonian given in the main text is diagonalized assuming that the HF-potential is periodic, i.e.

$$V^{\mathrm{HF}}_{\sigma\mathbf{k}}(\mathbf{q}) = \sum_{\mathbf{G}} v_{\sigma\mathbf{k}}(\mathbf{G})\delta_{\mathbf{q},\mathbf{G}}. \tag{1}$$

We will show that this ansatz leads to periodic eigenstates which in turn give a consistent periodic HF-potential. Moreover, we will discuss how the reciprocal lattice vectors $\{\mathbf{G}\}$ of the WC need to be chosen to create a self-consistent solution. In order to diagonalize the Hamiltonian, we first perform a zone folding,i.e. $\tilde{a}^{\dagger}_{\sigma\mathbf{k}\mathbf{G}} = a^{\dagger}_{\sigma\mathbf{k}+\mathbf{G}}$,, and restrict the summations over $\mathbf{k}$ to the first Brillouin zone of the WC. In the zone-folded frame, the HF-Hamiltonian reads

$$H = \sum_{\sigma\mathbf{k}\mathbf{G}} \Big(\tilde{\varepsilon}_{\sigma\mathbf{k}}(\mathbf{G})\tilde{a}^{\dagger}_{\sigma\mathbf{k}\mathbf{G}} + \sum_{\mathbf{G}'} \tilde{v}_{\sigma\mathbf{k}}(\mathbf{G},\mathbf{G}')\tilde{a}^{\dagger}_{\sigma\mathbf{k}\mathbf{G}'}\Big)\tilde{a}_{\sigma\mathbf{k}\mathbf{G}}, \tag{2}$$

where we have defined the abbreviations $\tilde{\varepsilon}_{\sigma\mathbf{k}}(\mathbf{G}) = \varepsilon_{\sigma\mathbf{k}+\mathbf{G}}$ and $\tilde{v}_{\sigma\mathbf{k}}(\mathbf{G},\mathbf{G}') = v_{\sigma\mathbf{k}+\mathbf{G}}(\mathbf{G}-\mathbf{G}')$. Hence, the Hamiltonian in Eq. (2) becomes diagonal via the basis transformation

$$\tilde{a}^{\dagger}_{\sigma\mathbf{k}\mathbf{G}} = \sum_{\lambda} u^{\lambda}_{\sigma\mathbf{k}}(\mathbf{G})A^{\dagger}_{\lambda\sigma\mathbf{k}}, \tag{3}$$

where the Bloch-wave coefficients have to fulfill the eigenvalue equation

$$\tilde{\varepsilon}_{\sigma\mathbf{k}}(\mathbf{G})u^{\lambda}_{\sigma\mathbf{k}}(\mathbf{G}) + \sum_{\mathbf{G}'} \tilde{v}_{\sigma\mathbf{k}}(\mathbf{G},\mathbf{G}')u^{\lambda}_{\sigma\mathbf{k}}(\mathbf{G}') = E^{\lambda}_{\sigma\mathbf{k}}u^{\lambda}_{\sigma\mathbf{k}}(\mathbf{G}). \tag{4}$$

This equation exactly represents the well-known Hartree-Fock equation, but here in momentum space, without any external potential of nuclei and assuming periodic boundary conditions. After performing the basis transformation. the Coulomb interaction is now absorbed into the eigenenergies of the system and we obtain

$$H = \sum_{\lambda\sigma\mathbf{k}} E^{\lambda}_{\sigma\mathbf{k}}A^{\dagger}_{\lambda\sigma\mathbf{k}}A_{\lambda\sigma\mathbf{k}}. \tag{5}$$

Now, we take a closer look at the HF-potential within the Wigner basis. From the symmetry of Eq. (4) follows $u^{\lambda}_{\sigma\mathbf{k}+\mathbf{G}'}(\mathbf{G}) = u^{\lambda}_{\sigma\mathbf{k}}(\mathbf{G}+\mathbf{G}')$ and consequently $A_{\lambda\sigma\mathbf{k}+\mathbf{G}} = A_{\lambda\sigma\mathbf{k}}$. Moreover, the HF-potential is determined by the density matrix $\langle a^{\dagger}_{\sigma\mathbf{k}+\mathbf{q}}a_{\sigma\mathbf{k}}\rangle$. After transformation into the Wigner basis, the temporal evolution of the new density matrix is determined by the diagonal Hamiltonian in Eq. (5). Therefore, off-diagonal elements have no source terms and will decay via dephasing, such that in the thermodynamic equilibrium it holds $\langle A^{\dagger}_{\lambda\sigma\mathbf{k}+\mathbf{q}}A_{\nu\sigma\mathbf{k}}\rangle = f^{\lambda}_{\sigma\mathbf{k}}\delta_{\lambda\nu}\sum_{\mathbf{G}}\delta_{\mathbf{q},\mathbf{G}}$. With the above relation, it is straightforward to show that within the WC basis the HF-potential takes the form of Eq. (1) with the Fourier components given by

$$v_{\sigma\mathbf{k}}(\mathbf{G}) = \sum_{\lambda\sigma'\mathbf{k}'\mathbf{G}'} f^{\lambda}_{\sigma'\mathbf{k}'}(V_{\mathbf{G}} - V_{\mathbf{k}'-\mathbf{k}+\mathbf{G}'}\delta_{\sigma\sigma'}) \times u^{\lambda *}_{\sigma'\mathbf{k}'}(\mathbf{G}'-\mathbf{G})u^{\lambda}_{\sigma'\mathbf{k}'}(\mathbf{G}'). \tag{6}$$

Finally, we need to determine the lattice geometry and the occupation numbers $f^{\lambda}_{\sigma\mathbf{k}}$. Both are consistently obtained with a *maximum distance ansatz* assuming that the electrons will maximize their interparticle distance to reduce the Coulomb repulsion. Hence, the crystal structure for electrons with the same spin is given by the hexagonal close pack, such that $\mathbf{G} = i\mathbf{G}_0 + jC_3\mathbf{G}_0$ where i and j are integers and C_3 is a rotation by 120°. The lattice parameter is then determined by the electron density assuming a single electron per unit cell and spin, i.e. $n = n_{\uparrow} + n_{\downarrow} = 2n_{\uparrow}$ and $n_{\sigma} = 1/A_{\mathrm{uc}} = 2/(\sqrt{3}a_0^2)$. Hence, the density determines the lattice structure via $a_0 = \sqrt{4/(\sqrt{3}n)}$ and $|\mathbf{G}_0| = 4\pi/(\sqrt{3}a_0)$, which at the same time fixes the occupation of the WC bands. Each band contributes exactly one momentum state per unit cell. Consequently, if the temperature is low enough, such that $k_BT \ll E^1_{\sigma\mathbf{k}} - E^0_{\sigma\mathbf{k}}$, the lowest band has to be fully occupied and the rest is empty ($f_{\lambda\sigma\mathbf{k}} = \delta_{\lambda 0}$) resulting in $n_{\sigma} = \dfrac{1}{A}\sum_{\lambda\mathbf{k}} f_{\lambda\sigma\mathbf{k}} = \dfrac{1}{(2\pi)^2}\int_{\mathrm{BZ}} d^2k = \dfrac{A_{\mathrm{BZ}}}{(2\pi)^2} = \dfrac{1}{A_{\mathrm{uc}}}$, as we initially claimed.

* brem@uni-marburg.de
† ermin.malic@chalmers.se

II. WIGNER CRYSTALS IN TMD MONOLAYERS

The Coulomb interaction between electrons in a TMD monolayer is well described by the generalized Rytova-Keldysh potential [3, 4]. Moreover, we account for the background charge created by the nuclei with the Jellium model, removing the interaction between electrons at $\mathbf{q} = 0$,

$$V_{\mathbf{q}} = \begin{cases} 0 & \text{for } \mathbf{q} = 0 \\ e_0^2\big(2\epsilon_0 A q \epsilon_s(q)\big)^{-1} & \text{else} \end{cases} \tag{7}$$

$$\epsilon_{\mathrm{s}}(q) = \kappa_{\mathrm{ml}} \tanh\left(\frac{1}{2}\left[\alpha_{\mathrm{ml}} dq - \ln\left(\frac{\kappa_{\mathrm{ml}} - \kappa_{\mathrm{bg}}}{\kappa_{\mathrm{ml}} + \kappa_{\mathrm{bg}}}\right)\right]\right) \tag{8}$$

with $\kappa = \sqrt{\epsilon^{\parallel}\epsilon^{\perp}}$ and $\alpha = \sqrt{\epsilon^{\parallel}/\epsilon^{\perp}}$ indicating the dielectric constants of the monolayer (ml) and the background (bg), respectively. For hBN-encapsulated samples, we use $\kappa_{\mathrm{bg}} = 4.5$ [5], whereas the effective dielectric background of a sample on SiO_2 is an average $\kappa_{\mathrm{bg}} = (3.9{+}1)/2$ [6]. All relevant material constants for the numerical implementation of Eq. (4) for WSe_2 and $MoSe_2$ monolayers have been summarized in Table I and stem from established literature [7, 8].

Table I. Relevant parameters for the computation of WC in TMD monolayers. Hole masses m_h are take from Ref. [7] in terms of the electron rest mass, whereas the monolayer thickness d and parallel/perpendicular dielectric constants are taken from Ref. [8]

Material	m_h $[m_0]$	d [nm]	$\epsilon^{\parallel}$	$\epsilon^{\perp}$
WSe_2	0.36	0.652	15.1	7.5
$MoSe_2$	0.6	0.645	16.5	7.4

We have developed a numerical code that finds the solution of Eq. (4) via iteration in a discretized $\mathbf{k}$-space and with a finite number of $\mathbf{G}$ vectors. In the beginning, the components of $u^0_{\sigma\mathbf{k}}(\mathbf{G})$ determining the HF-potential are randomized. With the resulting HF-potential the eigenvalues and -vectors of Eq. 4 are determined via a diagonalization algorithm. The eigenvectors are then fed back into the HF-potential and the procedure is repeated until the resulting energies and wave functions stop changing between iteration steps, i.e. a self-consistent solution is found. Depending on the electron density it can take up to 200 iterations for the algorithm to converge. The results shown in the main text are highly converged with respect to iterations as well as $\mathbf{k}$-space resolution and number of reciprocal lattice vectors. In particular, the excited states are converged with respect to their contribution to the optical response, i.e. the resolution of the numerical calculation is increased until the obtained response spectrum, such as displayed in Fig. 3 of the main text, does not show any visible changes anymore. This is achieved by including 20x20 k-points and the 187 smallest G-vectors (7 shells).

III. INTERACTION WITH LIGHT

Now, we determine the interaction Hamiltonian between electrons in the WC and classical light. Since we want to consider the interaction with low-frequency laser light with a perpendicular incidence to the monolayer we use the length gauge (dipole approximation). In perpendicular geometry the strength of the electric field $\mathbf{E}(t)$ does not depend on the position within the monolayer, such that the electron light Hamiltonian in plane wave basis reads

$$H_{\text{e-f}} = ie_0 \mathbf{E} \sum_{\sigma\mathbf{k}} a^{\dagger}_{\sigma\mathbf{k}} \nabla_{\mathbf{k}} a_{\sigma\mathbf{k}} \tag{9}$$

Next, we transform the electronic operators into the Wigner basis, which yields two contributions

$$\begin{aligned} H_{\text{e-f}} &= ie_0 \mathbf{E} \sum_{\lambda\sigma\mathbf{k}} A^{\dagger}_{\lambda\sigma\mathbf{k}} \nabla_{\mathbf{k}} A_{\lambda\sigma\mathbf{k}} \\ &+ \sum_{\lambda\lambda'\sigma\mathbf{k}} \mathbf{E} \cdot \mathbf{d}^{\lambda\lambda'}_{\sigma\mathbf{k}} A^{\dagger}_{\lambda\sigma\mathbf{k}} A_{\lambda'\sigma\mathbf{k}} \end{aligned} \tag{10}$$

with the dipole matrix element $\mathbf{d}^{\lambda\lambda'}_{\sigma\mathbf{k}} = ie_0 \sum_{\mathbf{G}} u^{\lambda}_{\sigma\mathbf{k}}(\mathbf{G}) \nabla_{\mathbf{k}} u^{\lambda'*}_{\sigma\mathbf{k}}(\mathbf{G})$. We find that the presence of the light field induces off-diagonal elements of the density matrix $\rho^{\lambda\lambda'}_{\sigma\mathbf{k}} = \langle A^{\dagger}_{\lambda\sigma\mathbf{k}} A_{\lambda'\sigma\mathbf{k}}\rangle$ (interband coherences). However, we consider the limiting case, in which the perturbation is weak enough, such that the change in the HF-potential can be neglected. We split off the electrical field induced change of the density matrix, i.e. $\rho^{\lambda\lambda'}_{\sigma\mathbf{k}} = \delta_{\lambda\lambda'}\delta_{\lambda,0} + \delta\rho^{\lambda\lambda'}_{\sigma\mathbf{k}}$, and compute its temporal evolution via the Heisenberg equation of motion [9, 10]. Neglecting all non-linearities we find

$$\begin{aligned} i\hbar\partial_t \delta\rho^{\lambda\lambda'}_{\sigma\mathbf{k}} &= (E^{\lambda'}_{\sigma\mathbf{k}} - E^{\lambda}_{\sigma\mathbf{k}} - i\Gamma)\delta\rho^{\lambda\lambda'}_{\sigma\mathbf{k}} \\ &+ (\delta_{\lambda,0} - \delta_{\lambda',0})\mathbf{E} \cdot \mathbf{d}^{\lambda'\lambda}_{\sigma\mathbf{k}} \end{aligned} \tag{11}$$

Here, we have added a phenomenological damping constant Γ, leading to a decay of interband coherences due to dephasing.

Next, we want to deduce a macroscopic observable, e.g. given by the electrical current in the system

$$\mathbf{j} = -\frac{e_o\hbar}{m_* A} \sum_{\sigma\mathbf{k}} \mathbf{k}\langle a^{\dagger}_{\sigma\mathbf{k}} a_{\sigma\mathbf{k}}\rangle = \frac{1}{A} \sum_{\lambda\lambda'\sigma\mathbf{k}} \mathbf{J}^{\lambda\lambda'}_{\sigma\mathbf{k}} \delta\rho^{\lambda\lambda'}_{\sigma\mathbf{k}} \tag{12}$$

with the current matrix element $\mathbf{J}^{\lambda\lambda'}_{\sigma\mathbf{k}} = -\frac{e_o\hbar}{m_*} \sum_{\mathbf{G}} u^{\lambda}_{\sigma\mathbf{k}}(\mathbf{G}) \mathbf{G} u^{\lambda'*}_{\sigma\mathbf{k}}(\mathbf{G})$. Using Eq. (4) and neglecting the weak exchange interaction, a relation between the current matrix element $\mathbf{J}^{\lambda\lambda'}_{\sigma\mathbf{k}}$ and the transition dipole matrix element $\mathbf{d}^{\lambda\lambda'}_{\sigma\mathbf{k}}$ can be derived, reading

$$\mathbf{J}^{\lambda\lambda'}_{\sigma\mathbf{k}} \approx \frac{i}{\hbar}(E^{\lambda'}_{\sigma\mathbf{k}} - E^{\lambda}_{\sigma\mathbf{k}})\mathbf{d}^{\lambda\lambda'}_{\sigma\mathbf{k}}. \tag{13}$$

Finally, combining Eq. (12) and (11) we can find an expression for the diagonal part of the linear optical conductivity via

$$\sigma(\omega) = \frac{\mathbf{j}(\omega)\cdot\hat{\mathbf{e}}}{d|\mathbf{E}(\omega)|} = \frac{i\hbar}{Ad}\sum_{\sigma\lambda\mathbf{k},\pm}\frac{|J^{\lambda}_{\sigma\mathbf{k}}|^2}{\Delta E^{\lambda}_{\sigma\mathbf{k}}}\Delta\tilde{E}^{\lambda}_{\sigma\mathbf{k}}(\omega)^{-1}, \quad (14)$$

where we have introduced the projected current $J^{\lambda}_{\sigma\mathbf{k}} = \hat{\mathbf{e}}\cdot\mathbf{J}^{\lambda,0}_{\sigma\mathbf{k}}$ onto the polarization vector $\hat{\mathbf{e}}$ of the electric field, the transition energy $\Delta E^{\lambda}_{\sigma\mathbf{k}} = E^{\lambda}_{\sigma\mathbf{k}} - E^{0}_{\sigma\mathbf{k}}$, and $\Delta\tilde{E}^{\lambda}_{\sigma\mathbf{k}}(\omega) = \hbar\omega \pm \Delta E^{\lambda}_{\sigma\mathbf{k}} + i\Gamma$. Note that the above conductivity corresponds to the linear response of the system to very weak perturbations.

[1] Trail, J.; Towler, M.; Needs, R. Unrestricted Hartree-Fock theory of Wigner crystals. *Physical Review B* **2003**, *68*, 045107.

[2] Pan, H.; Wu, F.; Sarma, S. D. Quantum phase diagram of a moiré-Hubbard model. *Physical Review B* **2020**, *102*, 201104.

[3] Rytova, N. Screened potential of a point charge in a thin film. *Moscow University Physics Bulletin* **1967**, *3*, 30.

[4] Brem, S.; Ekman, A.; Christiansen, D.; Katsch, F.; Selig, M.; Robert, C.; Marie, X.; Urbaszek, B.; Knorr, A.; Malic, E. Phonon-assisted photoluminescence from indirect excitons in monolayers of transition-metal dichalcogenides. *Nano Letters* **2020**, *20*, 2849–2856.

[5] Geick, R.; Perry, C.; Rupprecht, G. Normal modes in hexagonal boron nitride. *Physical Review* **1966**, *146*, 543.

[6] Robertson, J. High dielectric constant oxides. *The European Physical Journal-Applied Physics* **2004**, *28*, 265–291.

[7] Kormányos, A.; Burkard, G.; Gmitra, M.; Fabian, J.; Zólyomi, V.; Drummond, N. D.; Fal'ko, V. k·p theory for two-dimensional transition metal dichalcogenide semiconductors. *2D Materials* **2015**, *2*, 022001.

[8] Laturia, A.; Van de Put, M. L.; Vandenberghe, W. G. Dielectric properties of hexagonal boron nitride and transition metal dichalcogenides: from monolayer to bulk. *npj 2D Materials and Applications* **2018**, *2*, 1–7.

[9] Lindberg, M.; Koch, S. W. Effective Bloch equations for semiconductors. *Physical Review B* **1988**, *38*, 3342.

[10] Kira, M.; Koch, S. Many-body correlations and excitonic effects in semiconductor spectroscopy. *Progress in Quantum Electronics* **2006**, *30*, 155–296.
